# First reports of clinical effects of transjugular intrahepatic portosystemic shunt in four patients with cirrhotic ascites refractory to tolvaptan

**DOI:** 10.1136/bmjgast-2023-001120

**Published:** 2023-04-21

**Authors:** Kota Tsuruya, Jun Koizumi, Yuka Sekiguchi, Shun Ono, Tatsuya Sekiguchi, Takuya Hara, Yusuke Mishima, Yoshitaka Arase, Shunji Hirose, Koichi Shiraishi, Tatehiro Kagawa

**Affiliations:** 1 Division of Gastroenterology and Hepatology, Department of Internal Medicine, Tokai University School of Medicine, Isehara, Japan; 2 Department of Diagnostic Radiology, Tokai University School of Medicine, Isehara, Japan; 3 Department of Comprehensive Radiology, Chiba University Hospital, Chiba, Japan

**Keywords:** LIVER CIRRHOSIS, PORTAL HYPERTENSION, INTERVENTIONAL RADIOLOGY, ASCITES

## Abstract

**Objective:**

Ascites in patients with decompensated cirrhosis can lead to abdominal distention and decrease quality of life. Tolvaptan, a vasopressin V2 receptor antagonist, is an effective agent in the treatment of ascites, whereas some patients are refractory to tolvaptan. The efficacy of transjugular intrahepatic portosystemic shunt (TIPS) for these patients is not known. In this study, we performed TIPS for tolvaptan-refractory cirrhotic patients and analysed its efficacy and safety in these patients.

**Design:**

This retrospective analysis included patients with liver cirrhosis who received TIPS for ascites or hydrothorax refractory to tolvaptan therapy along with conventional diuretics between January 2015 and May 2018 at Tokai University Hospital. We evaluated the efficacy and safety of TIPS.

**Results:**

This study included four patients. All patients presented with Child-Pugh class B liver cirrhosis and model for end-stage liver disease-sodium scores were 10/12/14/16. TIPS was generated successfully without any major complications in all patients. The body weight decreased by a mean of 4.7 (SD=1.0) kg and estimated glomerular filtration rate improved from a mean of 38.2 (SD=10.3) to 59.5 (SD=25.0) mL/min/1.73 m^2^ in a month after TIPS procedure.

**Conclusion:**

TIPS is an effective potential treatment for ascites in patients with tolvaptan refractory condition. In appropriate patients who can tolerate TIPS, the treatment may lead towards renal function improvement.

**WHAT IS ALREADY KNOWN ON THIS TOPIC**

- Tolvaptan, a vasopressin V2 receptor antagonist, is an effective agent in the treatment of refractory ascites in cirrhosis, whereas some patients are refractory to tolvaptan.

**WHAT THIS STUDY ADDS**

- This study found that transjugular intrahepatic portosystemic shunt (TIPS) is an effective potential treatment for ascites in patients with tolvaptan refractory condition.
- The effect of TIPS reduced diuretics and may lead towards renal function improvement in these patient.

**HOW THIS STUDY MIGHT AFFECT RESEARCH, PRACTICE OR POLICY**

- Although next-line treatment options for ascites in tolvaptan-refractory cases are limited, TIPS is a treatment option in appropriate cirrhotic patients.

## Introduction

Ascites is a common symptom of decompensated cirrhotic patients and significantly impairs the quality of life due to abdominal distention. Ascites appears in approximately 5%–10% of compensated cirrhotic patients per year.^1^ Refractory ascites (RA), defined as ascites that does not recede by appropriate medical therapy such as salt restriction and diuretic therapy or recures shortly after large volume paracentesis (LVP),^2^ is associated with poor survival.^3^ According to the treatment guidelines for cirrhosis with RA issued by the American Association for the Study of Liver Diseases,^4^ the European Association for the Study of Liver diseases 5 and the Japan Society of Hepatology (JSH),^6 7^ additional therapy such as LVP with albumin infusion, cell-free and concentrated ascites refusion therapy (CART), peritoneovenous shunt, transjugular intrahepatic portosystemic shunt (TIPS) and liver transplantation should be considered.

TIPS is a well-established technique for reducing portal pressure and subsequently improves ascites.^8 9^ In addition, TIPS increases the cardiac output and decreases systemic vascular resistance, causing an effective reduction in circulating blood volume and improving renal perfusion.^10^ In clinical practice, TIPS can improve RA and prognosis,^11 12^ although success of TIPS creation depends on eligible patients and appropriate timing.^13^

Tolvaptan, a selective oral vasopressin V2 receptor antagonist, excretes water by inhibiting water reabsorption in the renal collecting tubule and exhibits diuretic activity without increase of electrolyte excretion. Tolvaptan has been used in clinical practice for hyponatraemia,^14 15^ heart failure^16^ and autosomal dominant polycystic kidney disease.^17^ In Japan, tolvaptan has been indicated for fluid retention in cirrhosis since September 2013, and its efficacy has been reported against RA.^18 19^ Guidelines from JSH recommend tolvaptan as a treatment option against RA. On the other hand, the efficacy of tolvaptan in cases of refractory fluid retention has been reported to be 50%–78%, and some cirrhotic patients do not respond to tolvaptan.^20^ As far as we know, RA cases who received TIPS after failure of tolvaptan therapy were very few. In this study, we investigated the efficacy and safety of TIPS in patients with RA who did not respond to tolvaptan.

## Materials and methods

### Patients and study design

We enrolled four consecutive patients with liver cirrhosis who received TIPS for RA or hydrothorax under tolvaptan therapy between January 2015 and May 2018 at Tokai University Hospital. These patients had received tolvaptan for RA that was poorly controlled by sodium restriction and administration of furosemide (≥20 mg/day) and spironolactone (≥25 mg/day), but their ascites did not control even with tolvaptan treatment and repeated LVP with albumin infusion and/or CART. Response to TIPS was defined as weight loss ≥1.5 kg on day 7 from baseline.^21^ We retrospectively analysed the background, drug history, laboratory data and clinical course until November 2022.

### TIPS procedure

All TIPS procedures were performed by an experienced interventional radiologist using conventional technique.^22^ First, a 10-French sheath was placed via the right femoral artery, and superior mesenteric artery portography and coeliac angiography were performed. The position of the hepatic artery and portal vein was confirmed on coeliac arterioportography, and a catheter was advanced to the right hepatic artery corresponding to the right portal branch approximately 2 cm from the porta hepatis as a landmark. Next, a 4-French sheath was placed via the right jugular vein. The Rosch-Uchida transjugular liver access set (Cook Medical, Bloomington, Indiana, USA) was used to create portal vein access and the tract was dilated using a Mustang 8 mm balloon catheter (Boston Scientific Corporation, Marlborough, Massachusetts, USA). The use of stents and the choice of stent device were decided by the radiologist. Self-expanding bare nitinol stents such as Luminex×12 mm×6 cm (Bard Peripheral Vascular, Tempe, Arizona, USA), Luminex×12 mm×8 cm (Bard Peripheral Vascular), Zilver 635 10 mm×8 cm (Cook Medical, Bloomington, Indiana, USA) and Epic vascular stent 10 mm×8 cm (Boston Scientific, Marlborough, Massachusetts, USA) were implanted. The pressures of the portal vein, right atriumand central vein were measured using a pressure transducer system. The portosystemic pressure gradient (PSG) was determined as the difference between the pressures in the portal vein and in the right atrium. ΔPSG was calculated by the difference in PSGs before and at the end of TIPS creation. TIPS is currently not covered by national health insurance in Japan, thus, TIPS procedure was planned and performed with sufficient informed consent.

## Results

### Clinical characteristics

**Figure 1:**
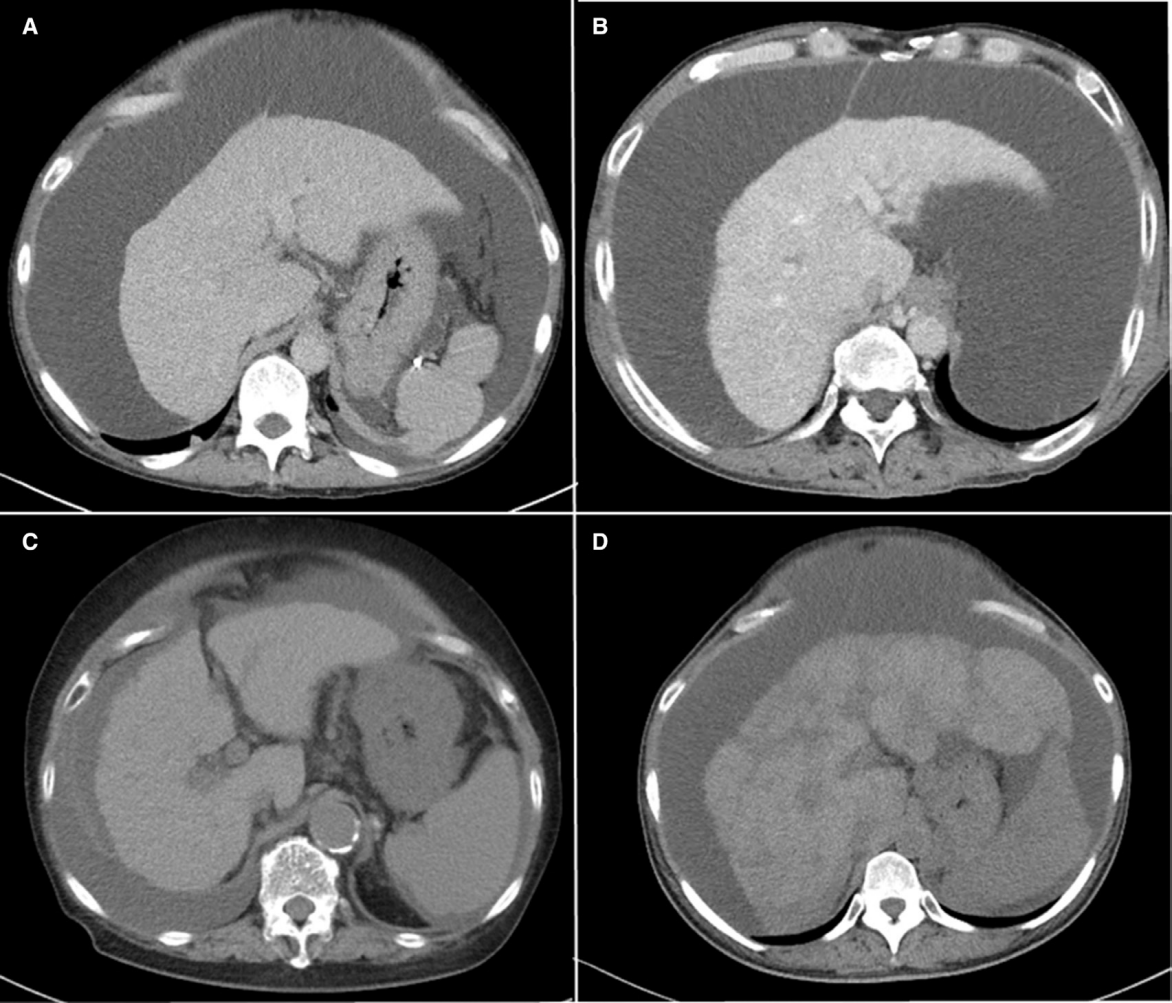
Abdominal computed tomography scan images before TIPS creation. (A) Case 1, (B) case 2, (C) case 3 and (D) case 4. TIPS, transjugular intrahepatic portosystemic shunt.

TIPS was performed in four patients with RA who did not respond to the addition of tolvaptan to the existing diuretics such as furosemide and spironolactone. General characteristics in the patients are described in table 1. Aetiology of liver diseases were hepatitis C virus, alcoholic liver disease, non-alcoholic steatohepatitis and sarcoidosis, one by one. All patients presented with Child-Pugh class B liver cirrhosis. Model for end-stage liver disease-sodium (MELD-Na) scores were 10/12/14/16. Concomitantly used diuretics were furosemide at doses of 20–80 mg, spironolactone at doses of 25–200 mg and tolvaptan at a dose of 7.5 mg. Figure 1 shows CT images of ascites and pleural effusion before TIPS.

**Table 1:** Clinical characteristics of the patients

	Case 1	Case 2	Case 3	Case 4
Sex	Male	Male	Female	Female
Age (years)	56	58	73	40
Aetiology	HCV	ALD	NASH	Sarcoidosis
Platelet (×10^4^/µL)	13.0	9.5	11.3	11.2
PT (%)	67	98	104	105
Alb (g/dL)	3.3	3.3	2.9	2.2
T-Bil (mg/dL)	1.7	1.1	0.3	0.5
Cre (mg/dL)	1.45	1.43	1.83	1.00
eGFR (ml/min/1.73 m^2^)	40.7	40.9	21.6	49.7
Child-Pugh score	9	8	8	9
MELD score	14	10	12	6
MELD-Na score	16	14	12	10
Spleno-renal shunt	–	–	–	–
HE history	–	–	–	–
Diuretics	Furosemide 40 mg Spironolactone 100 mg Tolvaptan7.5mg	Furosemide 80 mg Spironolactone 200 mg Tolvaptan 7.5 mg	Furosemide 20 mg Spironolactone 25 mg Tolvaptan 7.5 mg	Furosemide 60 mg Spironolactone 50 mg Tolvaptan 7.5 mg
Period between administration of tolvaptan and TIPS procedure (month)	9	10	6	3

Alb, albumin; ALD, alcoholic liver disease; Cre, creatinine; eGFR, estimated glomerular filtration rate; HCV, hepatitis C virus; HE, hepatic encephalopathy; MELD-Na, model for end-stage liver disease-sodium; NASH, non-alcoholic steatohepatitis; PT, prothrombin time; T-Bil, total bilirubin; TIPS, transjugular intrahepatic portosystemic shunt.

### TIPS clinical efficacy against ascites and complication

**Figure 2:**
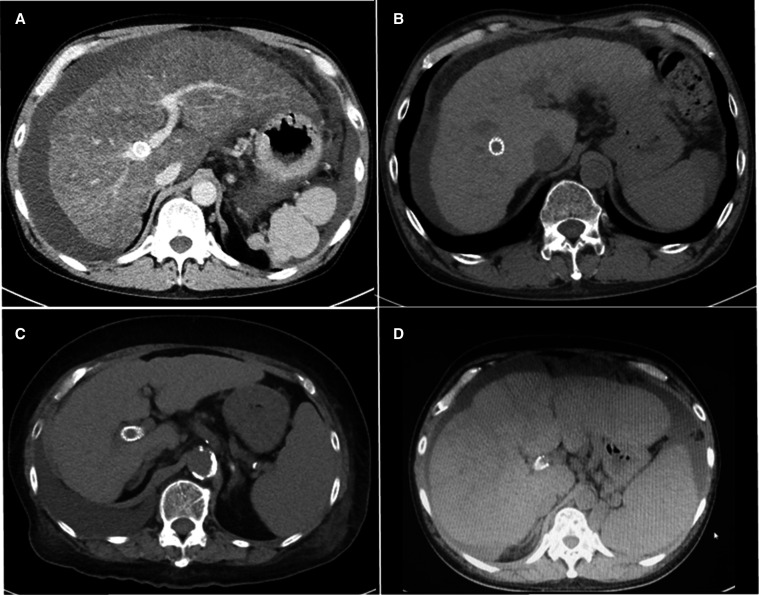
Abdominal computed tomography scan images post TIPS creation. (A) Case 1, (B) Case 2, (C) case 3 and (D) case 4. TIPS, transjugular intrahepatic portosystemic shunt.

TIPS was successfully created in all patients. The PSG decreased from a mean of 17.5 (SD=3.1) to 8.8 (SD=3.3) mmHg. All patients achieved response to TIPS along with the decrease in body weight by 4.7 (SD=1.0) kg in 1 month (table 2). As shown in figure 2, ascites and pleural fluid receded in all patients. The patients were prophylactically given rifaximin and hepatic encephalopathy was not observed. There were no deaths within the 30 days and no apparent complications associated with TIPS.

**Table 2:** Clinical course and outcome of the patient

	Case 1	Case 2	Case 3	Case 4
PSG pre-TIPS (mmHg)	16	15	17	22
PSG post-TIPS (mmHg)	12	7	5	11
ΔPSG (mmHg)	4	8	12	11
Stent diameter and length used in TIPS	12 mm×6 cm	12 mm×8 cm	10 mm×8 cm	10 mm×8 cm
Change in body weight at 1 month after TIPS (kg)	−4.2	−3.7	−6.1	−4.6
Serum creatinine levels at 1 month after TIPS (mg/dL)	0.65	1.29	1.33	0.80
eGFR at 1 month after TIPS (mL/min/1.73 m^2^)	97.9	45.8	30.6	63.5
Readmission due to HE post-TIPS	–	–	–	–
Diuretics after discharge	Furosemide 20 mg Spironolactone 50 mg Tolvaptan 7.5 mg	Furosemide 40 mg Spironolactone 100 mg Tolvaptan 7.5 mg	Furosemide 10 mg Spironolactone 25 mg Tolvaptan 7.5 mg	Furosemide 40 mg Spironolactone 125 mg Tolvaptan 7.5 mg

eGFR, estimated glomerular filtration rate; HE, hepatic encephalopathy; PSG, portosystemic pressure gradient; TIPS, trasnjugular intrahepatic portosystemic shunt.

### Renal function and diuretic dose post-TIPS procedure

In 1 month after TIPS procedure, the estimated glomerular filtration rate (GFR) improved in all patients with the increase from a mean of 38.2 (SD=10.3) to 59.5 (SD=25.0) mL/min/1.73 m^2^. Diuretics were adjusted after TIPS procedure due to improvement in fluid retention. Subsequently, in the outpatient setting, tolvaptan was continued in all patients, while the doses of furosemide were reduced (table 2).

### Mortality outcomes

Case 3 died of pneumonia; 83 days after TIPS, and case 1 died of unexplained sudden death; 112 days after TIPS. While, other two patients survived under ascites control.

## Discussion

The first treatment for fluid retention in cirrhosis is salt restriction followed by conventional diuretics such as spironolactone and loop diuretics.^6 7^ Increasing doses of diuretics such as furosemide activates the sympathetic nervous system and renin–angiotensin–aldosterone (RAA) system, decreases GFR and raises the risks of renal impairment, electrolyte abnormalities and sarcopenia.^23 24^ High furosemide administration doses may also decrease renal interstitial osmolarity, leading to refractoriness against tolvaptan.^25^ Tolvaptan exerts its action independently of serum albumin concentration and does not reduce the renal blood flow and GFR because it does not affect RAA system.^25–27^ There are also reports that the use of tolvaptan reduced furosemide doses in cases of chronic kidney disease stages 3–4.^28^ According to the JSH cirrhosis guidelines,^6 7^ from the viewpoint of preventing deterioration of renal function early add-on administration of tolvaptan is recommended for patient who are resistant to conventional diuretics such as spironolactone (25–50 mg/day) and furosemide (20–40 mg/day). In cases of ascites refractory to conventional diuretics, tolvaptan induction has been shown to be more effective than increasing the dose of furosemide.^29^ On the other hand, factors prior to tolvaptan induction such as high blood urea nitrogen value,^30^ low serum natrium value^31^ and decreased liver function (based on high Child-Pugh score)^32^ are predicted to decrease response to tolvaptan treatment.

Treatment for RA includes TIPS, serial LVP plus albumin infusion, PV shunts, low-flow ascites pumps and liver transplantation. TIPS is an endovascular operation which is performed to create an intrahepatic tract between the hepatic and portal veins and can reduce the portal pressure. Compared with serial LVP, TIPS reduced the relapse of symptoms due to ascites and improves transplant-free survival.^33^ Selection of eligible patients is important for the success of TIPS creation. Child-Pugh class C,^34^ MELD score greater than 18,^35^ advanced age,^33^ cardiopulmonary insufficiency or sarcopenia^36 37^ have a high risk for complications of TIPS creation. In addition, Bureau *et al* reported serum bilirubin value below 50 µmol/L and a platelet count above 75×10^9^/L is predictive of survival in patients with RA treated with TIPS.^38^ In this study, all patients were Child-Pugh grade B, MELD score was less than 18, and serum bilirubin and platelet count were within above criteria, indicating appropriate patient selection. We do not derive the exact mechanisms why these four patients responded to TIPS well. However, TIPS might be as effective in the tolvaptan-refractory patients as in the patients with RA as long as they fit the eligibility criteria of TIPS described above.

The larger diameter-stent can more reliably achieve the target hepatic venous pressure gradient. Whereas excessive shunting may induce hepatic dysfunction due to reduced effective hepatic blood flow and the development of encephalopathy. Therefore, it is important to select the appropriate diameter of the stent to balance the efficacy and complications of TIPS.^39^ A comparative study of 8 mm and 10 mm diameter stents showed no difference in the development of hepatic encephalopathy, but control of complications due to portal hypertension was superior with the 10 mm diameter stent.^40 41^ In this study, the stents used for two patients in TIPS placement were 10 mm and 12 mm each, which were effective in improving ascites and did not cause any hepatic encephalopathy or dysfunctions. This discrepancy is attributed to bare stents used in our country as different from a more popular stent-graft in Western countries. Because of more frequent restenosis of bare stents in TIPS, bigger stent diameters tends to be selected in our hospital.

This study shows, for the first time, the therapeutic results of TIPS in a patient refractory to tolvaptan with effective results. The effect of TIPS and the controllable ascites showed that renal function improved in this patient after TIPS due to the diuretics reduction, especially furosemide. Our study has limitations. This study is a retrospective, single-centre study with a small number of patients, which will include selection bias. The accumulation of cases is necessary to evaluate the safety and efficacy of TIPS in these patients. Next-line treatment options for ascites in tolvaptan-refractory cases are limited, but TIPS is a promising treatment option in appropriate cases. In conclusion, TIPS was clinically successful in the treatment of RA in tolvaptan-refractory patients.

## Data Availability

Data are available upon reasonable request.

## References

[1] Forns X, Ginès A, Ginès P, et al. Management of ascites and renal failure in cirrhosis. Semin Liver Dis 1994;14:82–96. 10.1055/s-2007-1007300 8016665

[2] Moore KP, Wong F, Gines P, et al. The management of ascites in cirrhosis: report on the consensus conference of the international ascites club. Hepatology 2003;38:258–66. 10.1053/jhep.2003.50315 12830009

[3] Moreau R, Delègue P, Pessione F, et al. Clinical characteristics and outcome of patients with cirrhosis and refractory ascites. Liver Int 2004;24:457–64. 10.1111/j.1478-3231.2004.0991.x 15482343

[4] Biggins SW, Angeli P, Garcia-Tsao G, et al. Diagnosis, evaluation, and management of ascites, spontaneous bacterial peritonitis and hepatorenal syndrome: 2021 practice guidance by the american association for the study of liver diseases. Hepatology 2021;74:1014–48. 10.1002/hep.31884 33942342

[5] Angeli P, Bernardi M, Villanueva C, et al. EASL clinical practice guidelines for the management of patients with decompensated cirrhosis. Journal of Hepatology 2018;69:406–60. 10.1016/j.jhep.2018.03.024 29653741

[6] Yoshiji H, Nagoshi S, Akahane T, et al. Evidence-based clinical practice guidelines for liver cirrhosis 2020. Hepatol Res 2021;51:725–49. 10.1111/hepr.13678 34228859

[7] Yoshiji H, Nagoshi S, Akahane T, et al. Evidence-Based clinical practice guidelines for liver cirrhosis 2020. J Gastroenterol 2021;56:593–619. 10.1007/s00535-021-01788-x 34231046 PMC8280040

[8] Richter GM, Palmaz JC, Nöldge G, et al. The transjugular intrahepatic portosystemic stent-shunt. A new nonsurgical percutaneous method. Radiologe 1989;29:406–11. 2798853

[9] Tripathi D, Stanley AJ, Hayes PC, et al. Transjugular intrahepatic portosystemic stent-shunt in the management of portal hypertension. Gut 2020;69:1173–92. 10.1136/gutjnl-2019-320221 32114503 PMC7306985

[10] Busk TM, Bendtsen F, Poulsen JH, et al. Transjugular intrahepatic portosystemic shunt: impact on systemic hemodynamics and renal and cardiac function in patients with cirrhosis. Am J Physiol Gastrointest Liver Physiol 2018;314:G275–86. 10.1152/ajpgi.00094.2017 29074483

[11] Narahara Y, Kanazawa H, Fukuda T, et al. Transjugular intrahepatic portosystemic shunt versus paracentesis plus albumin in patients with refractory ascites who have good hepatic and renal function: a prospective randomized trial. J Gastroenterol 2011;46:78–85. 10.1007/s00535-010-0282-9 20632194

[12] Salerno F, Merli M, Riggio O, et al. Randomized controlled study of tips versus paracentesis plus albumin in cirrhosis with severe ascites. Hepatology 2004;40:629–35. 10.1002/hep.20364 15349901

[13] Wong F, Sniderman K, Liu P, et al. Transjugular intrahepatic portosystemic stent shunt: effects on hemodynamics and sodium homeostasis in cirrhosis and refractory ascites. Ann Intern Med 1995;122:816–22. 10.7326/0003-4819-122-11-199506010-00002 7741365

[14] Schrier RW, Gross P, Gheorghiade M, et al. Tolvaptan, a selective oral vasopressin V2-receptor antagonist, for hyponatremia. N Engl J Med 2006;355:2099–112. 10.1056/NEJMoa065181 17105757

[15] Albert NM, Nutter B, Forney J, et al. A randomized controlled pilot study of outcomes of strict allowance of fluid therapy in hyponatremic heart failure (SALT-HF). J Card Fail 2013;19:1–9. 10.1016/j.cardfail.2012.11.007 23273588

[16] Imamura T, Kinugawa K. Update of acute and long-term tolvaptan therapy. J Cardiol 2019;73:102–7. 10.1016/j.jjcc.2018.10.003 30420105

[17] Torres VE, Chapman AB, Devuyst O, et al. Tolvaptan in patients with autosomal dominant polycystic kidney disease. N Engl J Med 2012;367:2407–18. 10.1056/NEJMoa1205511 23121377 PMC3760207

[18] Sakaida I, Kawazoe S, Kajimura K, et al. Tolvaptan for improvement of hepatic edema: a phase 3, multicenter, randomized, double-blind, placebo-controlled trial. Hepatol Res 2014;44:73–82. 10.1111/hepr.12098 23551935

[19] Bellos I, Kontzoglou K, Psyrri A, et al. Tolvaptan response improves overall survival in patients with refractory ascites: A meta-analysis. Dig Dis 2020;38:320–8. 10.1159/000503559 31578028

[20] Iwamoto T, Maeda M, Saeki I, et al. Analysis of tolvaptan non-responders and outcomes of tolvaptan treatment of ascites. J Gastroenterol Hepatol 2019;34:1231–5. 10.1111/jgh.14524 30370940

[21] Hiramine Y, Uojima H, Nakanishi H, et al. Response criteria of tolvaptan for the treatment of hepatic edema. J Gastroenterol 2018;53:258–68. 10.1007/s00535-017-1366-6 28664229

[22] Keller FS, Farsad K, Rösch J. The transjugular intrahepatic portosystemic shunt: technique and instruments. Tech Vasc Interv Radiol 2016;19:2–9. 10.1053/j.tvir.2016.01.001 26997084

[23] Ginés P, Arroyo V, Quintero E, et al. Comparison of paracentesis and diuretics in the treatment of cirrhotics with tense ascites. results of a randomized study. Gastroenterology 1987;93:234–41. 10.1016/0016-5085(87)91007-9 3297907

[24] Hanai T, Shiraki M, Miwa T, et al. Effect of loop diuretics on skeletal muscle depletion in patients with liver cirrhosis. Hepatol Res 2019;49:82–95. 10.1111/hepr.13244 30156741

[25] Mori T, Ohsaki Y, Oba-Yabana I, et al. Diuretic usage for protection against end-organ damage in liver cirrhosis and heart failure. Hepatol Res 2017;47:11–22. 10.1111/hepr.12700 26990144

[26] Nakanishi K, Mattson DL, Gross V, et al. Control of renal medullary blood flow by vasopressin V1 and V2 receptors. Am J Physiol 1995;269(1 Pt 2):R193–200. 10.1152/ajpregu.1995.269.1.R193 7631893

[27] Jujo K, Saito K, Ishida I, et al. Randomized pilot trial comparing tolvaptan with furosemide on renal and neurohumoral effects in acute heart failure. ESC Heart Fail 2016;3:177–88. 10.1002/ehf2.12088 27818782 PMC5071712

[28] Matsue Y, Suzuki M, Torii S, et al. Clinical effectiveness of tolvaptan in patients with acute heart failure and renal dysfunction. J Card Fail 2016;22:423–32. 10.1016/j.cardfail.2016.02.007 26915749

[29] Uojima H, Hidaka H, Nakayama T, et al. Efficacy of combination therapy with natriuretic and aquaretic drugs in cirrhotic ascites patients: a randomized study. World J Gastroenterol 2017;23:8062–72. 10.3748/wjg.v23.i45.8062 29259382 PMC5725301

[30] Sakaida I, Terai S, Nakajima K, et al. Predictive factors of the pharmacological action of tolvaptan in patients with liver cirrhosis: a post hoc analysis. J Gastroenterol 2017;52:229–36. 10.1007/s00535-016-1233-x 27379386 PMC5281662

[31] Arase Y, Kagawa T, Tsuruya K, et al. Impaired renal function may not negate the efficacy of tolvaptan in the treatment of cirrhotic patients with refractory ascites. Clin Drug Investig 2019;39:45–54. 10.1007/s40261-018-0714-5 PMC6510826 30284699

[32] Yamada T, Ohki T, Hayata Y, et al. Potential effectiveness of tolvaptan to improve ascites unresponsive to standard diuretics and overall survival in patients with decompensated liver cirrhosis. Clin Drug Investig 2016;36:829–35. 10.1007/s40261-016-0434-7 27405984

[33] Salerno F, Cammà C, Enea M, et al. Transjugular intrahepatic portosystemic shunt for refractory ascites: a meta-analysis of individual patient data. Gastroenterology 2007;133:825–34. 10.1053/j.gastro.2007.06.020 17678653

[34] Lebrec D, Giuily N, Hadengue A, et al. Transjugular intrahepatic portosystemic shunts: comparison with paracentesis in patients with cirrhosis and refractory ascites: a randomized trial. french group of clinicians and a group of biologists. J Hepatol 1996;25:135–44. 10.1016/s0168-8278(96)80065-1 8878773

[35] Schepke M, Roth F, Fimmers R, et al. Comparison of MELD, child-pugh, and emory model for the prediction of survival in patients undergoing transjugular intrahepatic portosystemic shunting. Am J Gastroenterol 2003;98:1167–74. 10.1111/j.1572-0241.2003.07515.x 12809844

[36] Tsien C, Shah SN, McCullough AJ, et al. Reversal of sarcopenia predicts survival after a transjugular intrahepatic portosystemic stent. Eur J Gastroenterol Hepatol 2013;25:85–93. 10.1097/MEG.0b013e328359a759 23011041

[37] Nardelli S, Lattanzi B, Torrisi S, et al. Sarcopenia is risk factor for development of hepatic encephalopathy after transjugular intrahepatic portosystemic shunt placement. Clin Gastroenterol Hepatol 2017;15:934–6. 10.1016/j.cgh.2016.10.028 27816756

[38] Bureau C, Métivier S, D’Amico M, et al. Serum bilirubin and platelet count: a simple predictive model for survival in patients with refractory ascites treated by tips. J Hepatol 2011;54:901–7. 10.1016/j.jhep.2010.08.025 21145798

[39] Qi XS, Bai M, Yang ZP. Selection of a tips stent for management of portal hypertension in liver cirrhosis: an evidence-based review. WJG 2014;20:6470. 10.3748/wjg.v20.i21.6470 24914368 PMC4047332

[40] Riggio O, Ridola L, Angeloni S, et al. Clinical efficacy of transjugular intrahepatic portosystemic shunt created with covered stents with different diameters: results of a randomized controlled trial. J Hepatol 2010;53:267–72. 10.1016/j.jhep.2010.02.033 20537753

[41] Miraglia R, Maruzzelli L, Tuzzolino F, et al. Transjugular intrahepatic portosystemic shunts in patients with cirrhosis with refractory ascites: comparison of clinical outcomes by using 8- and 10-mm PTFE-covered stents. Radiology 2017;284:281–8. 10.1148/radiol.2017161644 28121521

